# Fibromuscular Dysplasia Leading to Spontaneous Coronary Artery Dissection with Sudden Cardiac Arrest

**DOI:** 10.1155/2015/708409

**Published:** 2015-06-03

**Authors:** Ata Bajwa, Udit Bhatnagar, Amit Sharma, Hani El-Halawany, Randall C. Thompson

**Affiliations:** Saint Luke's Mid America Heart Institute, University of Missouri-Kansas City School of Medicine, Kansas City, MO, USA

## Abstract

A 30-year-old previously healthy female, who was six-week postpartum, experienced sudden collapse and tonic-clonic seizure. Emergency medicine services arrived at the scene and the patient was found to be in ventricular fibrillation. Advanced cardiovascular life support (ACLS) was initiated with return of spontaneous circulation. Afterwards, her initial EKG showed atrial fibrillation with rapid ventricular rate, ST elevation in leads II, III, and aVF, and ST depression in V2–V4. She was transferred to a tertiary care hospital where emergent angiogram was performed revealing obstruction of blood flow in the proximal and mid right coronary artery (RCA). A hazy and irregularly contoured appearance of the RCA was consistent with diagnosis of fibromuscular dysplasia. Subsequently, intravascular ultrasonogram (IVUS) was performed which confirmed the diagnosis of RCA dissection. Successful revascularization of the RCA was performed using two bare mental stents. After a complicated course in hospital, she was discharged in stable condition and did very well overall.

## 1. Introduction

Fibromuscular dysplasia has been frequently cited to have close association with spontaneous coronary artery dissection (SCAD) which is an increasingly recognized cause of acute coronary syndrome, especially in young women. Prompt diagnosis and treatment can improve survival in patients with SCAD which otherwise may prove fatal.

## 2. Case

A 30-year-old Caucasian female presented after a sudden collapse followed by seizure activity that was witnessed by her husband. It was reported that she had woken up early in the morning and noticed abnormal sensations in both arms before collapsing. She had no significant past medical history and had spontaneous vaginal delivery of a healthy term infant, six weeks prior. On arriving at the scene, emergency medicine services found her in ventricular fibrillation. She was successfully defibrillated and taken to a nearby critical care hospital. Her initial EKG showed atrial fibrillation with rapid ventricular rate, ST segment elevation in the inferior leads, and ST depression in the anterior precordial leads. The ST elevation resolved spontaneously on subsequent EKGs, but ST depression persisted. She could not be airlifted because of inclement weather and, after few hours delay, was transferred to a tertiary care hospital via ambulance. Therapeutic hypothermia was initiated at the outlying hospital prior to transfer and was continued per protocol at our facility. Her serum troponin rose to 17.5 ng/mL. After her transfer, coronary angiogram was performed emergently.

**Figure 1:**
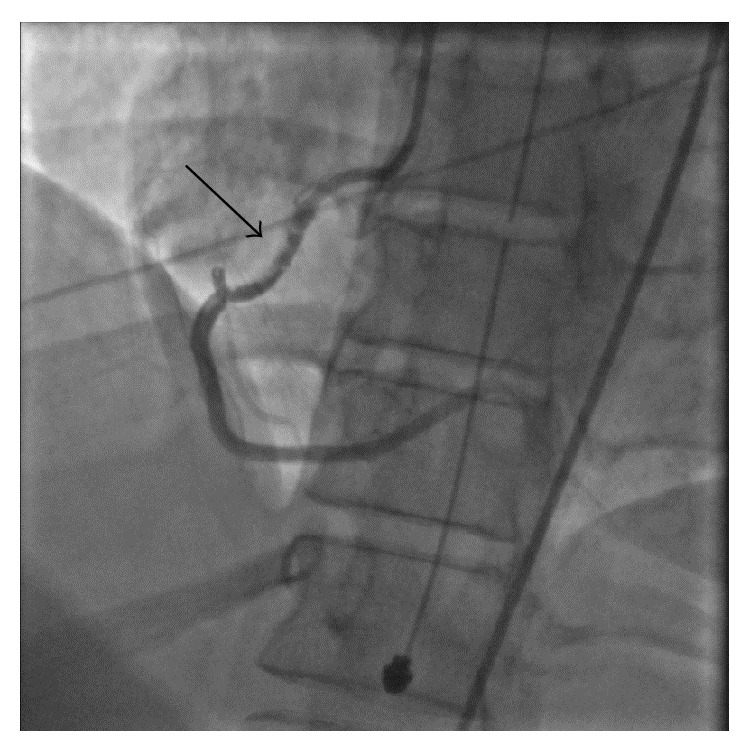
Coronary angiogram was performed within few hours after onset of symptoms and it showed 70% stenosis in proximal RCA flow. The lesion was irregularly contoured and hazy, which was consistent with fibromuscular dysplasia leading to spontaneous coronary artery dissection.

**Figure 2:**
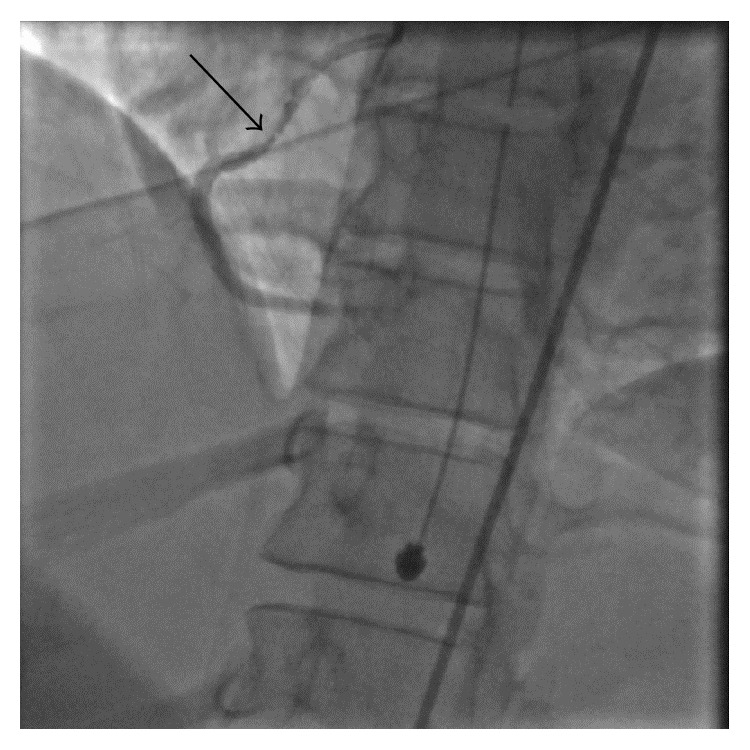
Stenosis seen in RCA became progressively worse after IC NTG and progressed to 99% occlusion. IVUS was performed and demonstrated coronary artery dissection.

Coronary angiography, as evident in Figure 1, showed 70% stenosis in the proximal and mid right coronary artery (RCA). The lesion was irregularly contoured and hazy and became more severe after intracoronary nitroglycerin was administered, progressing to 99% occlusion with TIMI II flow (Figure 2). Worsening of stenosis following nitroglycerin administration was considered to be inconsistent with coronary vasospasm. The angiographic appearance, young age of the patient, and lack of traditional risk factors for atherosclerotic disease were all consistent with a diagnosis of fibromuscular dysplasia. Coronary intravascular ultrasound (IVUS) was performed following angiography and showed dissection as well as thrombus in right coronary artery. The left main, left anterior descending, and circumflex coronary artery segments were angiographically normal.

**Figure 3:**
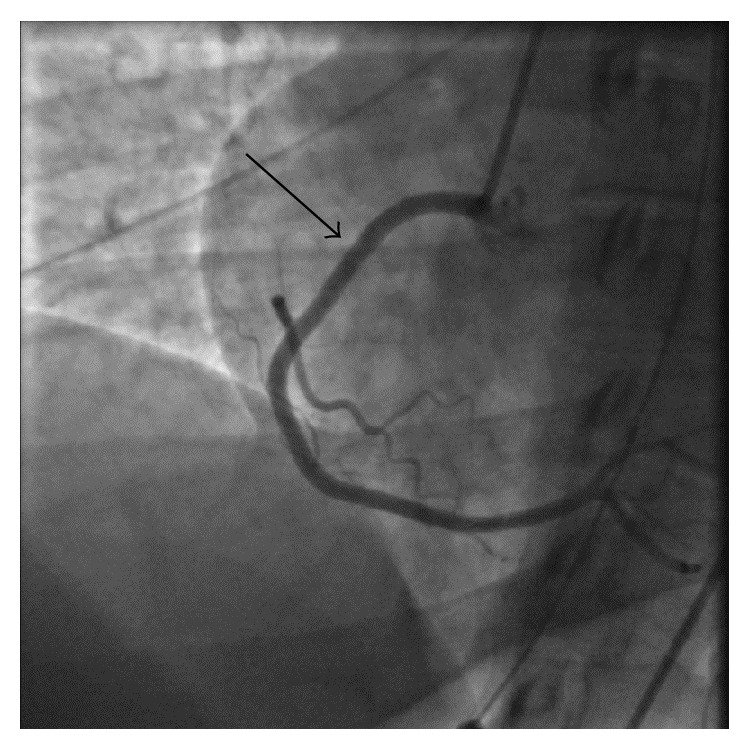
Emergent PCI was performed with placement of bare metal stents in proximal and mid RCA leading to restoration of the normal coronary blood flow (TIMI 3 flow).

The patient had originally presented after suffering cardiac arrest and she remained persistently hypotensive after being transferred to our facility. Because of the severe and worsening nature of the stenosis and the patient's hemodynamic instability, it was decided that IVUS directed coronary stent implantation would be appropriate. 3.5 mm × 30 mm and 3.5 × 15 mm integrity bare metal stent (Medtronic Corp) were implanted into the proximal right coronary artery and dilated to 14 atm. TIMI 3 flow was seen in RCA after the procedure (Figure 3). Optimal poststent implantation and expansion were confirmed with IVUS. Bare metal stents were chosen instead of drug eluting ones primarily because the patient had a very low predicted rate of restenosis based on our decision support algorithm. The low predicted rate was largely driven by the large caliber of the right coronary artery and the individual patient characteristics. The patient was comatose at the time of the procedure and limited medical history was available and there was some concern about bleeding risk with prolonged dual platelet therapy which would be necessary if drug eluting stents were used. She was started on aspirin 81 mg, ticagrelor 90 mg BID, intravenous heparin drip, and atorvastatin 80 mg QHS. Later on, metoprolol was also added after her blood pressure had improved.

An echocardiogram that was performed on the day of admission demonstrated severely reduced left ventricular systolic function with an estimated ejection fraction of 25%. There was inferior, inferolateral, and anterolateral left ventricular akinesis with near global hypokinesis. Right ventricular systolic function was normal. The patient's LV dysfunction was believed to be from cardiac ischemia/stunning related to a transient occlusion of the right coronary artery and the cardiac arrest. The patient had a complicated course over the next two weeks primarily related to anoxic brain injury from the cardiac arrest. She demonstrated myoclonic seizures and cortical blindness and was diagnosed with “Lance-Adams syndrome” and “Posterior Reversible Leukoencephalopathy syndrome,” respectively. She was started on a combination of antiepileptic medications resulting in improvement of her myoclonic seizures. A repeat echocardiogram was performed two weeks after the initial presentation, which demonstrated that regional and global left ventricular function had returned to normal. Her discharge medications included aspirin 81 mg, ticagrelor 90 mg BID, and metoprolol ER 25 mg. Atorvastatin was discontinued given that she had nonatherosclerotic disease and her LDL was 44 with a total cholesterol of 102. At time of discharge, she had some residual cortical blindness but improved markedly over the course of the next few months and ultimately made a complete neurological recovery including cessation of seizure activity.

## 3. Discussion

Fibromuscular dysplasia is an idiopathic nonatherosclerotic, noninflammatory vascular disease that primarily involves renal and carotid arteries, although coronary and other arterial vasculature systems can also be affected [1]. The etiology of FMD is uncertain, but it has been postulated that hormonal, genetic, metabolic, and traumatic factors might have a role [2]. About 90 percent of cases are diagnosed in women which could suggest some hormonal etiology. Estrogen thus may play a role, but there is no concrete evidence regarding this possibility. A genetic association has also been suggested and, according to some reports, about 7% to 11% of first-degree relatives have FMD. It has been hypothesized that genetically abnormal encasement of the vasa vasorum by connective tissue leads to medial ischemia, proliferation of myofibroblasts, and weakening of vessel wall thus rendering them vulnerable to dissection [3]. Fibromuscular dysplasia has been found to have a close association with dissection of arteries and may be the main predisposing factor for spontaneous coronary artery dissection (SCAD) [4]. Saw et al. studied 50 patients with nonatherosclerotic SCAD over a period of six years and concluded that as many as 86% of patients diagnosed with SCAD have FMD [4]. Another relatively small study by Toggweiler and colleagues published in 2012 also supported this association and suggested that patients diagnosed with SCAD should undergo screening for FMD [5].

Spontaneous coronary artery dissection (SCAD) is a rare pathologic condition that usually presents as acute coronary syndrome (ACS) or sudden cardiac death. The first case was reported in 1931, when a coronary artery dissection was found in a 42-year-old woman during an autopsy [6]. The incidence is much more common during pregnancy and the postpartum period, with SCAD reportedly responsible for 27% of cases of myocardial infarction (MI) in pregnant or postpartum women. This rate is significantly higher than in the general population where it accounts for only 0.28–1.1% of MI cases [7]. The pathophysiology of SCAD is not fully understood, though it is postulated that an intimal tear disrupts the vessel wall leading to true and false lumens [4]. An alternative postulated mechanism is that rupture of the vasa vasorum results in the formation of intramural hematoma [4]. Eventually, the expansion of the false lumen or hematoma can cause occlusion of the true vessel lumen leading to myocardial ischemia [4]. The mortality for SCAD in earlier studies was described to be as high as 50%, but, over the years, survival has improved to 85% owing to better availability of coronary diagnostic and treatment modalities [8].

Coronary angiography is the most widely used test and should not be delayed if a diagnosis of SCAD is suspected. The angiographic findings in SCAD can be quite variable, from multiple and extensive dissections seen in an otherwise normal appearing coronary artery to a single short dissection in a vessel with severe atherosclerosis [9]. Invasive angiography has its limitations though and sometimes may not distinguish between atherosclerotic and nonatherosclerotic lesions [10]. Intravascular ultrasound (IVUS) can directly visualize the vessel wall and may be used during angiography to help differentiate between true and false lumens [10]. Optical Coherence Tomography (OCT) has higher resolution and is superior to IVUS in determining double lumen morphology, intimal tear location, and extent of dissection [9]. There is no consensus on the specific guidelines for management of SCAD. Management decisions should be based on the hemodynamic status of the patient, the extent of dissection, the degree of vascular stenosis, and the adequacy of coronary flow [9]. If a patient is asymptomatic with limited vascular stenosis and adequate coronary flow, a conservative approach is appropriate and the patient can be medically treated with aspirin, beta blockers, and IV heparin [9]. Medical management in such cases usually leads to natural healing of the dissection over the course of next few months [9]. Thrombolytic drugs are usually avoided since they can potentially lead to worsening of dissection as well as intramural hematoma [10]. However, atherosclerosis is a much more common cause of acute coronary syndrome than SCAD, and therefore thrombolytic therapy should not be withheld for patients with ST elevation myocardial infarction (STEMI) in remote centers where primary percutaneous coronary intervention (PCI) is not available [10]. A more aggressive approach is reasonable if the patient is hemodynamically unstable or if the dissection leads to severe vascular stenosis (70–99% occlusion), resulting in inadequate coronary blood flow [9]. Such cases may require coronary revascularization through PCI, though it comes with risk of progression of the dissection and thus total arterial occlusion [9, 10]. Bioresorbable vascular scaffolds are being touted as an important new advance in interventional cardiology. They seem to work by transient scaffolding of the vessel wall while eluting an antiproliferative drug to counteract constrictive remodeling and intimal hyperplasia. Over the long term, the scaffolds get resorbed into the vessel wall resulting in enlarged vessel lumen and diminished atherosclerotic plaque [11]. Recently, the use of vascular scaffolds has been expanded for the treatment of nonatherosclerotic lesions including SCAD. Cockburn and colleagues recently reported a case of successful treatment of SCAD involving the left circumflex artery with excellent long term results [12]. There seems to be promise regarding their use in the management of SCAD, but more studies will be needed to strengthen this argument. Moreover, lack of availability and high cost may be limiting factors, at least for the time being. Coronary artery bypass grafting is usually reserved for cases involving left main coronary artery, multivessel dissection, and patients with failed PCI [9].

The patient in our case report presented after suffering a cardiac arrest as a result of RCA dissection. She underwent PCI and ultimately did well. Timely intervention can prove to be crucial for the survival of such critically ill patients. Patients diagnosed with SCAD should be assessed individually for decisions regarding medical management versus need for coronary revascularization.
